# Charge density wave memory in a cuprate superconductor

**DOI:** 10.1038/s41467-019-09433-1

**Published:** 2019-03-29

**Authors:** X. M. Chen, C. Mazzoli, Y. Cao, V. Thampy, A. M. Barbour, W. Hu, M. Lu, T. A. Assefa, H. Miao, G. Fabbris, G. D. Gu, J. M. Tranquada, M. P. M. Dean, S. B. Wilkins, I. K. Robinson

**Affiliations:** 1 Condensed Matter Physics and Materials Science Department, Brookhaven National Laboratory, Upton, NY 11973 USA; 2 National Synchrotron Light Source II, Brookhaven National Laboratory, Upton, NY 11973 USA; 3 Center for Functional Nanomaterials, Brookhaven National Laboratory, Upton, NY 11973 USA; 4 London Centre for Nanotechnology, University College, Gower St., London, WC1E 6BT UK; 5 Present Address: Materials Sciences Division, Lawrence Berkeley National Laboratory, Berkeley, CA 94720 USA; 6 Present Address: Stanford Synchrotron Radiation Lightsource, SLAC National Accelerator Laboratory, Menlo Park, CA 94025 USA

## Abstract

Although CDW correlations are a ubiquitous feature of the superconducting cuprates, their disparate properties suggest a crucial role for pinning the CDW to the lattice. Here, we report coherent resonant X-ray speckle correlation analysis, which directly determines the reproducibility of CDW domain patterns in La_1.875_Ba_0.125_CuO_4_ (LBCO 1/8) with thermal cycling. While CDW order is only observed below 54 K, where a structural phase transition creates inequivalent Cu-O bonds, we discover remarkably reproducible CDW domain memory upon repeated cycling to far higher temperatures. That memory is only lost on cycling to 240(3) K, which recovers the four-fold symmetry of the CuO_2_ planes. We infer that the structural features that develop below 240 K determine the CDW pinning landscape below 54 K. This opens a view into the complex coupling between charge and lattice degrees of freedom in superconducting cuprates.

## Abstract (web summary)

The understanding of charge density wave (CDW) correlations in cuprate superconductors remains hampered due to the lack of scattering phase information. Here, Chen et al. discover a reproducible CDW domain memory effect upon repeated cycling to temperatures well above the CDW ordering temperature.

## Introduction

Holes doped into the Mott insulating parent compounds of the high-temperature superconducting cuprates experience strong interactions with the antiferromagnetic background and with each other, as well as with the lattice in which they reside^1^. The Hubbard Hamiltonian, often used to model the cuprates, predicts that charge density wave (CDW) fluctuations are an intrinsic property of strongly interacting electrons in pristine, undistorted 2D square lattices^2,3^. CDWs have indeed been observed in essentially all hole-doped cuprates, but with distinct transition temperatures, correlation lengths, and wavevectors^4–11^. This is epitomized by comparing two very similar cuprates that have slightly different low-temperature crystal structures: La_1.875_Sr_0.125_CuO_4_ (LSCO 1/8) and LBCO 1/8. LSCO 1/8 has weak CDW correlations and is a bulk superconductor below its transition temperature of *T*_c_ = 28 K^8,12–14^; whereas LBCO 1/8 has well-correlated CDW order that almost completely suppresses bulk superconductivity into what is proposed to be a 2D superconducting pair density wave state^15–19^. As the main difference between these compounds is a subtle change in the crystal structure^20^, it is evident the lattice that hosts the CDW correlations has a dramatic influence on the properties of the CDW and the superconducting ground state.

Multiple types of lattice deformation or disorder are present in cuprates including that of interstitial oxygen atoms, chemical substitutions, as well as local and long-range tilting of the Cu–O octahedra, all of which have been proposed as possible CDW pinning features that stabilize CDW order^4,21–27^. One widely discussed lattice deformation in this context is the LTT phase in LBCO, in which octahedral tilts define a preferential direction for stripe pinning within each CuO_2_ plane^4,20,22^. While the LTO phase transition is common for both LBCO and LSCO, the LTT structural transition is a special feature in LBCO that coincides with the CDW formation. Fully understanding this process is, however, hampered by a lack of experimental techniques that are sensitive to the spatial distribution of the CDW order parameter.

This article reports our implementation of coherent resonant X-ray speckle correlation analysis as a tool for probing CDW domain pinning in the cuprates, choosing LBCO 1/8 as the model system due to its LTT structure and particularly large CDW correlation length. We discover strikingly reproducible CDW domain formation upon repeated thermal cycling well above its transition temperature and show that the CDW pinning memory is defined by structures that form at the LTO transition at 236(5) K rather than disorder or the LTT transition at 54(1) K that appears alongside CDW order.

## Results

### Experimental setup

**Fig. 1:**
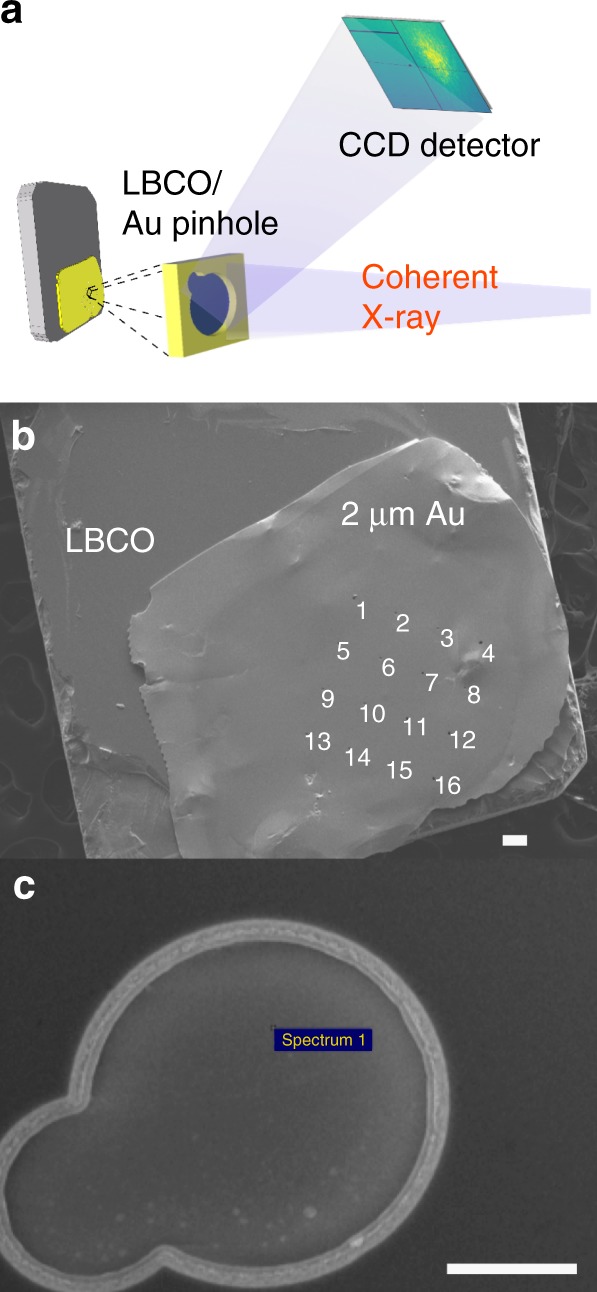
Experimental setup. **a** The scattering geometry in which coherent X-rays illuminate the masked LBCO 1/8 sample and the CDW Bragg peak is measured on a CCD detector. **b** A SEM image of the LBCO 1/8 single crystal with a Au mask fixed on top. Numbers 1–16 indicate the 16 pinholes drilled in the Au mask using a focused ion beam (FIB) prior to being fixed on the crystal. The white scale bar represents 50 μm. **c** A zoomed SEM image of pinhole #10, through which most of our data were taken. The white scale bar represents 5 μm

Although CDW pinning has potential to dramatically change the superconducting ground state, directly observing CDW pinning and tracking its changes with temperature are very challenging tasks. Much of the difficulty is that one must combine a technique that is sensitive enough to detect the CDW domain spatial arrangement, which we will call “texture”, with the ability to reproducibly illuminate the same sample volume over a wide temperature range. Figure 1 shows how we have addressed the problem using coherent resonant X-ray diffraction: a scattering method that measures the interference between scattering from different domains in the CDW texture as a “speckle” pattern. Our innovation, reported here, is to attach a mask with a microscopic pinhole to the sample, which we overfill with the coherent X-ray beam to ensure we illuminate the same sample volume despite possible temperature-induced drifts of the sample position.

CDWs in cuprates modulate the in-plane valance charge density with a period of ~3–4 lattice units along the Cu–O bond direction, and tend to stack out-of-phase from plane-to-plane leading to CDW propagation vectors of (*H*, 0, 0.5) or (0, *K*, 0.5), where *H* = *K* ≈ 0.2–0.35. For these experiments we chose an LBCO 1/8 single crystal which has one of the strongest CDW intensities of any cuprate and aligned it to its Bragg condition at wavevector **Q** = (0.236, 0, 1.5) (see Methods). We furthermore tuned the X-ray energy to the Cu *L*_3_-edge resonance around 931 eV in order to enhance our sensitivity to the weak CDW. In this condition the incident X-ray angle is *θ*_i_ = 88° with respect to the [001] sample surface and the detector angle is 2*θ* = 119°. Such an approach has been used extensively to study the average CDW properties^5–9,28–30^. The very high coherent flux (10^13^ photons/s) at the 23-ID-1 beamline at the National Synchrotron Light Source II (NSLS II) opens up the possibility of observing coherent interference between the domains of a weakly scattering order parameter, such as that of cuprate CDWs^31,32^. This was configured to produce a beam of ~20 μm at the sample overfilling the 10 μm pinhole in the Au mask attached to the sample (see Methods).

### Pinned domains within the ordered state

**Fig. 2:**
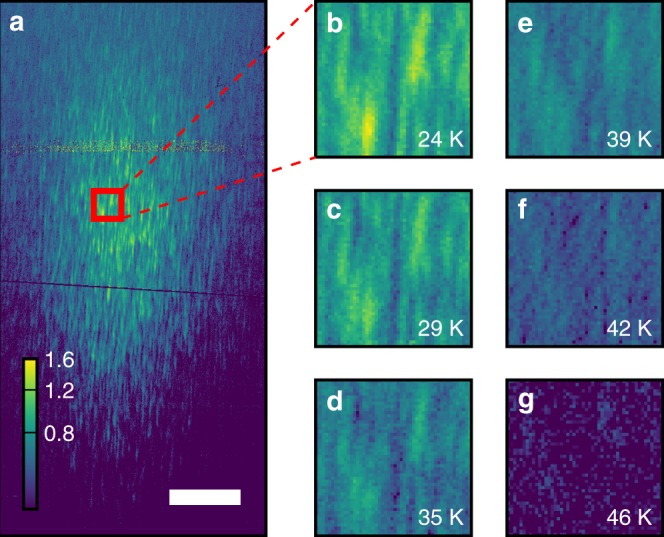
Temperature dependence of the CDW speckle positions within the ordered state. **a** A detector image of the LBCO 1/8 CDW Bragg peak at 24 K at **Q** = (0.236, 0, 1.5). The color-bar denotes intensity in photons s^−1^, and the white bar at the bottom indicates 100 detector pixels (0.0025 r.l.u). **b**–**g** The temperature dependence of speckle positions as the temperature was raised from 24 to 46 K. Zoomed-in speckle images are taken from the same detector area indicated by the red box in (**a**). Despite the broadening and weakening of the CDW Bragg peak, the speckles tend to persist in similar locations to 46 K, above which the speckle intensity becomes too low and noise dominates the signal

Figure 2a plots a detector image at the CDW Bragg condition, which is zoomed in panels b–g. The extent of the observed peak (in this case ~4 × 10^−3^ Å^−1^) is inversely proportional to the CDW correlation length (or domain size) similar to what is seen in conventional scattering. The speckle modulations on top of the peak envelope arise from coherent interference between different CDW domains. The average size and elongated shape of the speckles on the detector is determined primarily by the geometry of the experiment and the extent of the illuminated sample volume, which is fixed by the 10 μm pinhole and ~100 nm X-ray penetration depth (Fig. 1a) (see refs. ^31,32^ for a detailed discussion of the speckle shape and speckle visibility). Therefore, while the full speckle distribution contains information about the real-space domain distribution, the average speckle size and shape do not immediately provide information on the sample properties. The speckle locations, however, are highly sensitive to the CDW domain positions and can therefore be used to test for changes in the CDW domain texture as a function of temperature^33–35^. We note that while phase retrieval algorithms can in-principle reconstruct real-space images, the complexity of the domain pattern in this case makes this process intractable in practice^36^.

**Fig. 3:**
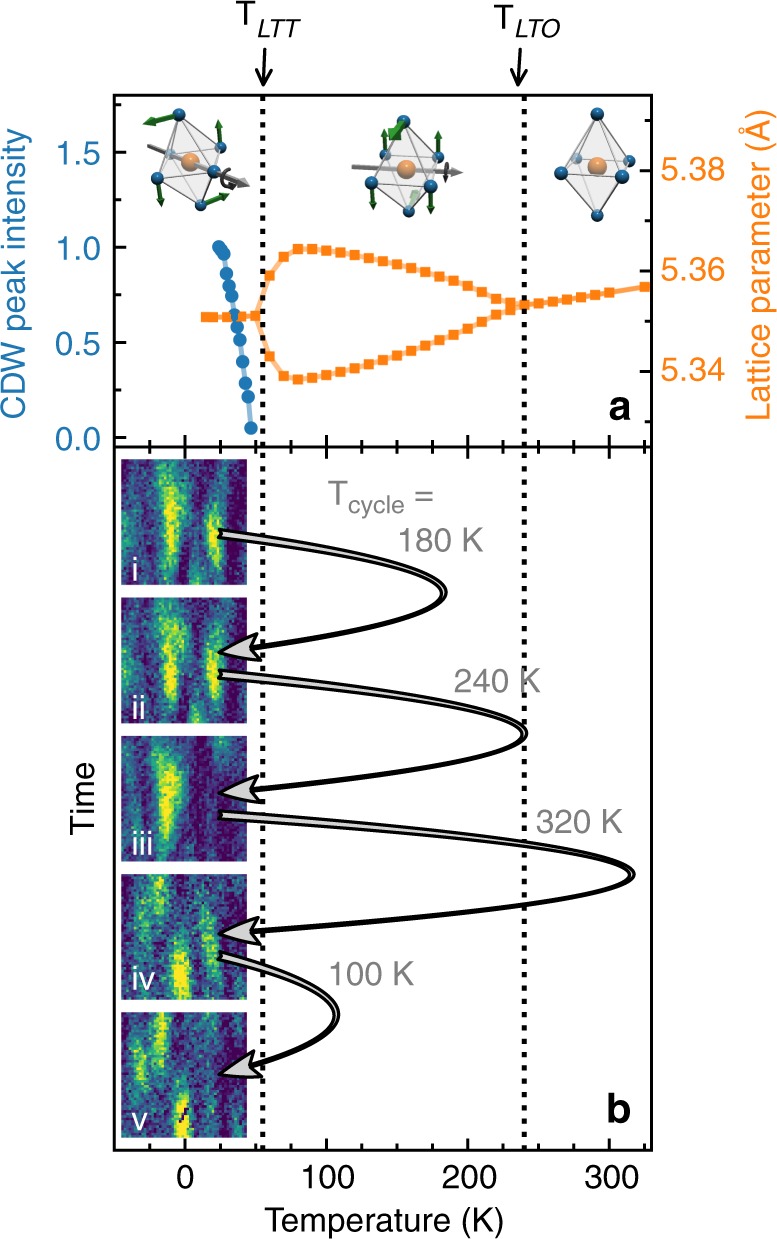
CDW and structural phases in LBCO 1/8 compared to speckle memory. **a** Temperature dependence of normalized CDW peak intensity (blue) and lattice parameters reproduced from ref. ^49^ (orange). The CDW transition coincides with the LTT structural transition at *T*_LTT_. A second lattice transition occurs at *T*_LTO_. The inset images represent the octahedral tilts associated with the LTT, LTO, and HTT phases. **b** CDW speckle images taken at 24 K. The black arrows indicate the temperature cycling between images. *T*_cycle_ indicates the highest temperature that sample was brought to during a temperature cycle

In LBCO 1/8, static CDW order exists at low temperatures with a correlation length of 240 Å. This can be directly verified with X-rays by noting that the speckle pattern does not vary as a function of time as reported previously^31,32^. With increasing temperature the CDW correlation length decreases and the CDW Bragg peak disappears coincident with *T*_LTT_ = 54 K^28,29,31,37–39^. (The temperature dependence of the CDW peak intensity is explicitly compared with the LTT to LTO structural phase transition in Fig. 3a later in the manuscript.) We measured a range of temperatures from 24 up to 46 K (the highest temperature for which we have sufficient CDW signal in order to observe the speckle locations). At all temperatures, the speckle locations were found to persist as illustrated in Fig. 2b–g.

### CDW domain memory

Speckle images were taken at 24 K successively before and after the sample temperature was cycled to *T*_cycle_ as we illustrate in Fig. 3b (see Supplementary Fig. 7 for a larger field of view). After cycling to *T*_cycle_ = 180 K, a temperature well above the CDW transition temperature, the speckle patterns are strikingly similar [see panels (i) and (ii)]. However, as the sample was heated to higher temperatures, the degree of reproducibility dropped, and by *T*_cycle_ = 320 K, their positions completely changed as seen in panels (ii) to (iv).

In order to compare speckle positions quantitatively, and identify the onset temperature of this change, we calculated the normalized cross-correlation function. Speckle images were background subtracted as described in Supplementary Note 1. These images are then represented as *M* × *N* matrices *A*_*m*,*n*_ and *B*_*m*,*n*_ where *m* and *n* are row-column indices. Cross-correlations matrices are calculated via

1 $$A_{m,n} \ast B_{m,n} = \mathop {\sum}\limits_{m{\prime} = - M}^M {\mathop {\sum}\limits_{n{\prime} = - N}^N {A_{m{\prime},n{\prime}}} } B_{m + m{\prime},n + n{\prime}}.$$

Here, *A*_*m*,*n*_ and *B*_*m*,*n*_ were taken to be *M* = 200 × *N* = 200-pixel images under the CDW peak measured before and after thermally cycling the sample, which include ~30–50 speckles. When *A*_*m*,*n*_ and *B*_*m*,*n*_ have the same or similar speckle patterns, the correlated intensity features a peak that we sum over to obtain a single normalized speckle cross-correlation coefficient^33–35^

2 $$\xi = \frac{{\mathop {\sum}\limits_{{\mathrm{speckle}}} {A_{m,n}} \ast B_{m,n}}}{{\left( {\mathop {\sum}\limits_{{\mathrm{speckle}}} {A_{m,n}} \ast A_{m,n}\mathop {\sum}\limits_{{\mathrm{speckle}}} {B_{m,n}} \ast B_{m,n}} \right)^{1/2}}}.$$

**Fig. 4:**
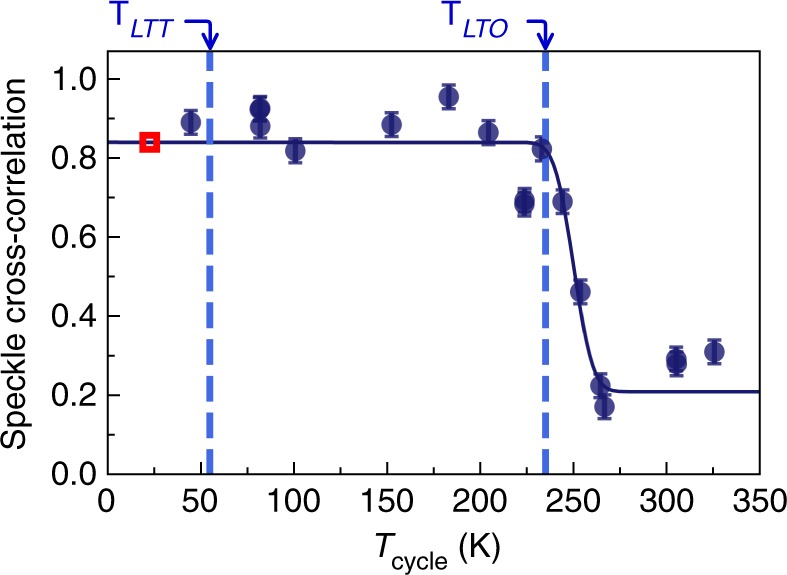
CDW memory. Normalized speckle cross-correlation coefficient, *ξ*, of data before and after temperature cycling to different values of *T*_cycle_. Images of 200 × 200 detector pixels were used for this calculation. An error function fit gives a de-correlation onset temperature of 240(3) K, which coincides with the LTO structural phase transition. The red box indicates 24 K the temperature where all the speckle images were sampled for this figure. Error bars are estimated by taking the standard deviation of repeated, nominally equivalent, measurements

In this equation, $\mathop {\sum}\nolimits_{{\mathrm{speckle}}}$ denotes summing over the peak in the cross-correlation matrix, corresponding to just over one speckle (~4 × 16 pixels) in size. The value is normalized by dividing by the auto-correlations of *A*_*m*,*n*_ and *B*_*m*,*n*_, such that two identical images have *ξ* = 1. In Fig. 4, we show *ξ* for various cycle temperatures showing a transition from high to low speckle reproducibility well above the CDW ordering temperature. We fit *ξ* versus *T*_cycle_ using an error function, which provides a reasonable phenomenological description of the shape of the transition. The onset temperature of de-correlation was 240(3) K, which coincides with the LTO structural phase transition at *T*_LTO_ = 236(5) K^37^. This transition involves rotations of the Cu–O octahedra around the 〈110〉_HTT_ direction and since nearest-neighbor octahedra rotate in opposite directions, the unit cell volume increases by a factor of two, with $a_{\mathrm{o}} \approx b_{\mathrm{o}} \approx \sqrt 2 a$. Below this threshold the majority of the speckles reproduce with *ξ* = 0.84(9); above the transition *ξ* = 0.21(4), which corresponds to the value expected in a random domain distribution (Supplementary Note 2). We further confirmed that different regions within the speckle pattern shown in Fig. 2a and data taken through another pinhole reproduce the same phenomenology within error bars.

## Discussion

These data establish that the CDW domains are strikingly reproducible upon temperature cycling well above the LTT and CDW transitions. The CDW is pinned at low temperatures, which is the usual behavior for a system with a long CDW correlation length as this can be accomplished by even a weak pinning force. This is, however, not a priori certain and the CDW in chromium has been reported to be dynamic down to low temperatures^40^. It further appears that the reproduciblity threshold here is associated with the LTO transition. This differs from what might have been anticipated based on the fact that the CDW appears at the LTT transition temperature^4,29,38,41^, which would suggest the reproduciblity threshold should match this temperature. We go on to discuss this intriguing result.

An analysis of hard X-ray Bragg diffraction data suggests that the typical LTO domain width is ~700 nm (Supplementary Note 3); we expect the LTT domain width to be comparable. In these experiments we illuminate a ~10 × 10 μm^2^ area of the sample with coherent X-rays, which will contain of order 200 LTT domains. All the CDW signal from this area contributes to the interference that determines the speckles. Within a given CuO_2_ layer, only half of the twin domains will contribute to a given CDW diffraction peak because of the unidirectional ordering within the LTT structure; the other half of the twin domains will contribute to a CDW peak rotated by 90°, i.e. at (0, 0.23, 1.5) rather than (0.23, 0, 1.5). Hence, in order for the speckle pattern to reproduce after cycling through the LTO-LTT transition, the domain pattern must reproduce precisely.

Now, the speckle pattern reproduces with cycling, but only if we use *T*_cycle_ < 240(3) K. This temperature matches the LTO–HTT transition. So if we cycle into the HTT phase, the resulting CDW speckle pattern in the LTT phase changes. This indicates that LTO domain boundaries play a crucial role in propagating the CDW domain memory.

How does the CDW domain memory created at LTO–HTT transition translate through LTT? Based on strain considerations, LTO domain boundaries are expected to occur along the 〈100〉_HTT_ lattice directions, as confirmed by TEM imaging^42–44^. Early structural studies of LBCO demonstrated that the LTT and LTO tilt patterns can be conceptualized as superpositions of one-another^20,45^. Such superposition-based arguments naturally justified the observation that LTT-like tilts occur at LTO domain boundaries in the cuprates^42–44^. Moreover, this supports the idea that LTT twin boundary locations can be inherited from the LTO twin boundary configuration. From the experimental results and the discussion above, we infer that the LTO twin domain pattern must change in a significant way on cycling into the HTT phase and that this process limits the observed CDW memory effect.

This is a remarkable result, but it is just the start. One might have expected a random nucleation of CDW domains on each cycling, which would change the speckles. The CDW order, with a period of ~4*a*, has a four-fold degeneracy with respect to how it aligns within a CuO_2_ layer of the LTT structure. The experiment, on the other hand, shows that the phase of the CDW order with respect to the lattice must be reproduced precisely each time. The CDW correlation length is just 24 nm (at low temperature), which is much smaller than the structural domain size. That implies that defects within a structural domain must locally pin the phase of the CDW order. Disorder and structural distortions are present in almost all cuprates and have been discussed extensively in connection with CDW pinning in the cuprates^4,21–27,46^. In La_2−*x*_Ba_*x*_CuO_4_ the most prominent form of disorder is La/Ba cation substitution, with locations that are independent of temperature. This may act in concert with the domain boundaries to pin the local CDW phase.

Another significant observation is that, within the CDW phase, the speckle locations do not change with temperature (see Fig. 2), despite the fact that the CDW correlation length varies with temperature^10,29,37,38,41^. This can be explained by uniform expansion of CDW domains around their pinning centers on cooling, as speckle locations are primarily dependent on the locations of the domains and largely insensitive to their size (Supplementary Note 4).

Almost all cuprates show some form of orthorhombic structural symmetry breaking, so the CDW memory effects discovered here may well be shared by many different cuprate species. Testing whether other cuprates, including those without a LTT phase, show similar behavior, and whether lattice pinning and the stability of CDW ordering are related will be important in future work. We further emphasize that the experimental configuration presented here, in which sample drift problems are avoided by attaching a mask directly to the sample, is also applicable to applied current or laser excitation, as well as to the magnetic field-dependent behavior studied previously in magnetic alloys^33–35^. This complements what can be achieved with scanning tunneling spectroscopy, which has superior spatial resolution, but is limited to cleaved surfaces^23^. The use of coherence and resonant X-ray scattering further opens the possibility to study charge, spin and orbital order parameters in other quantum materials with better resolution than is possible with current microdiffraction techniques^26^.

In conclusion, we present the first application of coherent resonant soft X-ray speckle correlation analysis to study the CDW domain hysteresis in LBCO 1/8. We uncover remarkably reproducible CDW domain formation upon temperature cycling far above the 54 K CDW transition, before the CDW pattern is almost completely reconfigured by cycling above 240(3) K. The CDW order, which is associated with the dramatic suppression of superconductivity in this sample, experiences a pinning landscape that is determined by structural domains that form at the LTO phase transition. Our results open a new route to study the complex interplay between lattice and charge degrees of freedom in quantum materials at cryogenic temperatures.

## Methods

### Sample preparation

Single crystals of LBCO 1/8 were grown using the floating zone method, and characterized extensively in previous studies^10,11,29,39,47^, all indicating excellent sample quality. A 2 μm gold (Au) film was evaporated onto a free-standing 200 nm Si_3_N_4_ membrane. Arrays of 10 μm asymmetric pinholes were drilled into the film using a focused ion beam yielding the shape shown in Fig. 1c. Although any stable shape is adequate for speckle correlation analysis, we chose to make a two-lobe structure. This decision was taken to break circular symmetry, which should improve the prospects for obtaining real-space images via Bragg coherent diffractive imaging in the future. Such attempts have thus far been unsuccessful. This assembly was pressed onto the LBCO single crystal with poly methyl methacrylate (PMMA) as a glue. The excess PMMA that filled in the pinholes was then removed using reactive ion etching. The resulting sample was imaged in a JEOL 7600F scanning electron microscope (Fig. 1b, c). In this paper, we index the crystal using the HTT unit cell where *a* = *b* = 3.78 and *c* = 13.28 Å. Correlation length here is defined as *a*/HWHM where HWHM is the half width at half maximum of the peak in reciprocal lattice units (r.l.u.).

### Coherent X-ray scattering

Data were taken at the Coherent Soft X-Ray (CSX) 23-ID-1 beamline at the NSLS II which is optimized to deliver high coherent flux at the sample. The beam was energy-dispersed in the vertical plane with a grating and focused onto a 20 μm pinhole ~5 mm from the sample to which the Au mask with a 10 μm pinhole was attached. In this configuration, the transverse coherence of the beam is greater than the extent of the illuminated region of the sample and the longitudinal coherence of the beam is of order 2 μm. The X-ray energy was further tuned to the Cu *L*_3_ absorption edge around 931 eV to enhance the signal from the CDW peak. The positions of the pinholes were determined by scanning the sample through the X-ray beam using the LBCO Cu fluorescent yield. We typically performed multiple measurements at each temperature to ensure that the cryostat was fully stable during the data collection. It typically required ~1 h to fully stabilize the cryostat at each temperature. Data were collected using a fast CCD^48^ with a 30 × 30 μm^2^ pixel size placed 340 mm from the sample. Images were read out every 5 s for a total collection time of ~10 min. A temperature ramp rate of 3 K min^−1^ was used for all the temperature cycles with an average waiting time of 5 min at the cycling temperature.

## Supplementary information

Supplementary Information

Source Data

## Data Availability

The data that support the findings of this study are available from the corresponding authors upon reasonable request.
