# Supplementary material for: Charge density wave memory in a cuprate superconductor
Source: Nat Commun. 2019 Mar 29;10:1435. doi: 10.1038/s41467-019-09433-1 (PMC6440992; doi:10.1038/s41467-019-09433-1)
Supplement: Supplementary file 1 — Supplementary Information [file 41467_2019_9433_MOESM1_ESM.pdf]

Supplementary Information

(Dated: March 20, 2019)

Supplementary Note 1.

In this section, we discuss the background treatment of the raw speckle data in order to obtain Fig. 4 in the main text and the fitting procedure to determine the onset of the transition. Our approach is similar to that used in previous x-ray speckle measurements of magnetic domains [1–3]. In our experiment, Cu L_3-edge resonant coherent scattering from LBCO 1/8 has three components: fluorescent background, the overall charge density wave (CDW) Bragg peak, and speckles arising from interference between different CDW domains [4, 5]. (See Fig. 2 of Ref [4] for an illustration of the decomposition of the different peak components.) The cross-correlation of two matrices A and B of size $M \times N$ is defined in Eq. 1 of the main text. Since A and B are two dimensional (2D) matrices, so is the result of the cross-correlation. In our calculation, we chose A (the matrix before the temperature cycle) and B (the matrix after the temperature cycle) to be 200×200 detector pixels, corresponding to the central area of the CDW peak. Example data measured at 24 K after cycling to $T_{\text{cycle}} = 224$ K is shown in Supplementary Figure 1(a)&(b). All data in Figs. 3 and 4 of the main text were collected at 24 K after cycling to different temperatures. The cross-correlation of the raw images (Supplementary Figure 1(a)&(b)) is plotted in Supplementary Figure 2(a), which is dominated by a triangular-shape resulted from fluorescent background and the overall CDW Bragg peak on top of the speckle signals. The triangular shape is more visible with a vertical line-cut plotted as the blue line in Supplementary Figure 2(c). In order to isolate speckles from background and incoherent CDW peak, we subtracted a smoothed envelope (*i.e.* $A^* = A - S(A)$, where S is a smoothing function) from raw A and B. Supplementary Figure 2 (b), and its line-cut in (c, green) displays the effect of this speckle isolation. This data treatment is necessary such that the cross-correlation calculation represents the reproducibility of the CDW domains independent of the overall background. For completeness we show the treated detector images in Supplementary Figure 1(c)&(d).

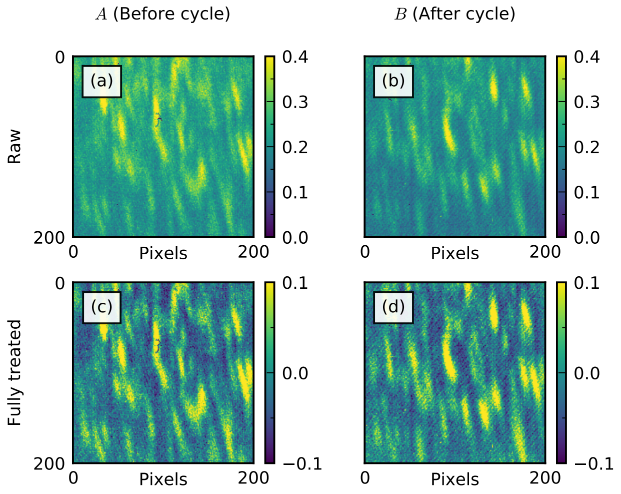

Supplementary Figure 1. Example raw and treated data taken at 24 K before and after a temperature cycle to $T_{\text{cycle}} = 224$ K corresponding to matrices A and B. (a)&(b) Raw data (a) before and (b) after the temperature cycle in units of photons s^{-1}. (c)&(d) Data after treatment (c) before and (d) after the temperature cycle.

After calculating the normalized cross-correlation coefficients for all the data points in Fig. 4 (main text), we used an error function

$$\text{erf}\,(x) = \frac{a}{\sqrt{\pi}} \int_0^{\frac{-(x-b)}{d}} e^{-t^2} dt + \frac{1}{2} + c \qquad (1)$$

to fit the shape of the transition. In this form, a, b, c and d are the step amplitude, center, offset and width, respectively. The t parameter is a dummy variable that is integrated out of the final form. The constants are chosen such that $\text{erf}(x)$ is a step function from 1 to 0 when $a = 1$, $b = 0$, $c = 0$ and $d \to 0^+$. We define the onset temperature

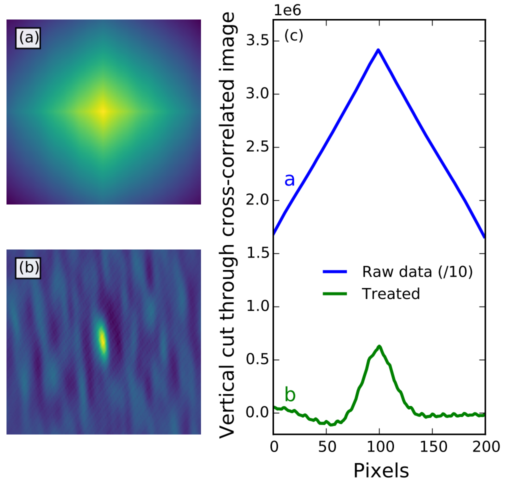

Supplementary Figure 2. (a),(b) Cross-correlation matrices of (a) raw data, (b) smoothed background subtracted fully treated data. (c) Vertical line cuts through the center of the cross-correlation matrices in (a) and (b).

of the transition as the intercept of two linear lines extrapolated from the slopes of the error function before and at the center-point of the transition, as shown in Supplementary Figure 3. We note that the height of the fit line on the left hand side of the plot may appear too low upon initial inspection, but this is due to the low values of the cross correlation at $T_{\mathrm{cycle}} = 223.6$ and 223.7 K, which fall well below the fit line.

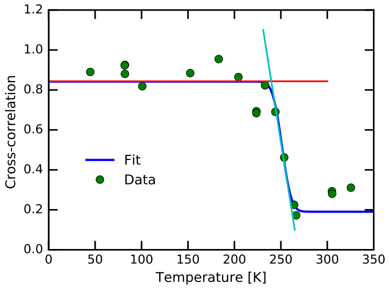

Supplementary Figure 3. Determination of the decorrelation onset temperature. The red and cyan lines are extrapolated from the low temperature limit and the midpoint of the error function, respectively. The onset is defined as the intersection of these two lines.

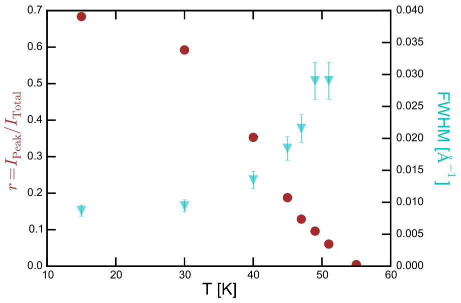

Supplementary Figure 4. The ratio of CDW peak intensity to the total intensity (brown circles, left axis) and FWHM (cyan inverted triangles, right axis) as a function of temperature obtained from fitting a Lorentzian-squared line shape to line cuts through the peak. Data were obtained from the same sample that we studied in the present paper [4]. The errorbars represent the 1σ uncertainty determined from the diagonal of the covariance matrix of the least-squares fitting routines.

Supplementary Note 2.

In order to estimate the cross-correlation for two random domain distributions, we have calculated the cross-correlation, ξ, of two simulated speckle patterns. Speckles were generated using sinc^2 functions with the same average width (4×16 pixels) and number density (~ 50 speckles in 200×200 detector pixels) as was measured and described in the main manuscript. We applied the same background subtraction routine, and obtained a $\xi = 0.19(6)$ after 100 trials. In addition, we computed ξ after flipping one of the 200×200 CDW detector images about a horizontal axis, and also obtained a similar ξ in its error range.

Supplementary Note 3.

In view of the relationship between the low temperature orthorhombic (LTO) and CDW domains, we measured the average LTO domain size by x-ray diffraction. A small crystal fragment of the same LBCO 1/8 sample was taken to the 34-ID-C beamline of the Advanced Photon Source (APS). A portable cryostat was used to cool the sample into the LTO phase at 140 K and the $(\frac{1}{2}\frac{1}{2}2)_{\mathrm{HTT}}$ (or $(012)_{\mathrm{LTO}}$) reflection was located on a Timepix detector with $p = 55$ μm pixels placed $D = 2.2$ m away. Because the 9 keV beam ($\lambda = 0.137$ nm) was coherent, the crystal was rocked in θ through the peak and the middle 30 frames (0.001° step size) were averaged together to smooth out the fringes. Radial and transverse cross sections were made through the detector diffraction pattern to extract the peak widths as shown in Supplementary Figure 5.

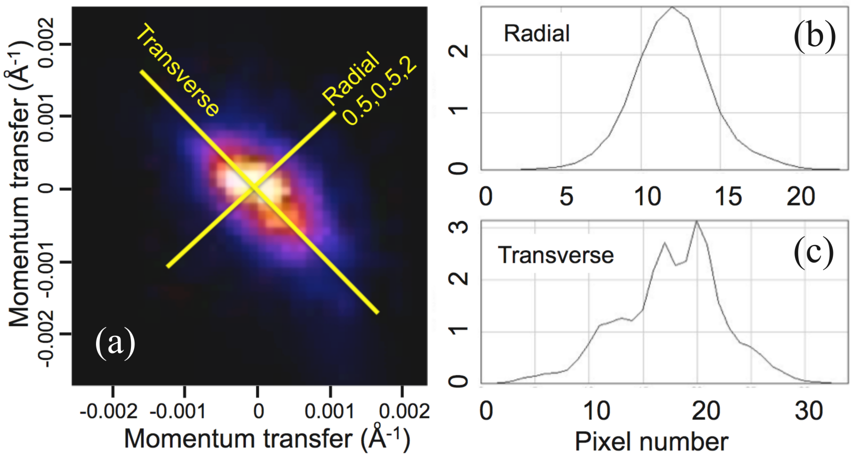

Supplementary Figure 5. Determination of LTO domain sizes. (a) Diffraction pattern of the $(\frac{1}{2}\frac{1}{2}2)_{\mathrm{HTT}}$ or $(012)_{\mathrm{LTO}}$ peak measured on a Timepix area detector. Profiles of the peak are shown in the (b) radial and (c) transverse directions.

The radial FWHM is 6 pixels wide, corresponding to an LTO domain size of $\lambda D/6p = 920$ nm along the $(\frac{1}{2}\frac{1}{2}2)_{\mathrm{HTT}}$ direction. The transverse FWHM is 13 pixels wide, giving a domain size of 420 nm. From the $\Delta\theta = 0.06^\circ$ FWHM width of the θ-scan (not shown), the domain size is $\lambda/2\sin\theta\Delta\theta = 400$ nm along the out-of-detector-plane direction. Here we take the average value of 700 nm as a typical LTO domain size. This number agrees well with the transmission electron microscopy (TEM) observations [6, 7].

Supplementary Note 4.

In Fig. 2 (main text), we saw persistent speckle positions when the temperature was varied in the low temperature tetragonal (LTT) phase. This observation might not be immediately intuitive because the average CDW domain size decreases with increasing temperature as seen in Supplementary Figure 4, so one might have expected to see a change in the speckle pattern. In this section, we calculate the speckle patterns of a simple artificial domain configurations to understand our observation. In the simulation, 500 domains were created at random positions within a circular area of diameter 600 pixels that represents the pinhole in the experiment. Each domain i at location (x_i, y_i) was

represented by a 2D Gaussian function

$$g(x,y) = \frac{1}{\sigma\sqrt{2\pi}} \exp\left(-\frac{(x-x_i)^2 + (y-y_i)^2}{2\sigma}\right) \tag{2}$$

where σ is the domain size. In this section, we compare the speckle patterns that arise from domain configurations with the same domain locations, but different domain sizes. Supplementary Figure 6 displays the result, in which a zoomed area of 100×100 pixels is shown. Panel (a) shows the initial configuration in which the domains were randomly assigned sizes of $\sigma = 4 \pm 2$ pixels. We found that very similar speckle locations are generated even if the domain size is doubled to $\sigma = 8 \pm 4$ as illustrated in Supplementary Figure 6(b)&(e). The difference in the speckle pattern with increasing domain size occurs mainly in the shape and distribution of the speckles, rather than their locations. This symmetric expansion of domains about the same locations explains the phenomenology seen in Fig. 2 (main text) where similar speckle patterns are measured despite a factor two change in correlation length as reported in Supplementary Figure 4. This applies up to the point at which domains merge as evidenced by the different patterns in Supplementary Figure 6(e)&(f). We note that in diffraction we only see one of two possible domains, so one does not necessarily expect to see merging domains, which will in any case be suppressed as adjacent CDW domains may have different phases.

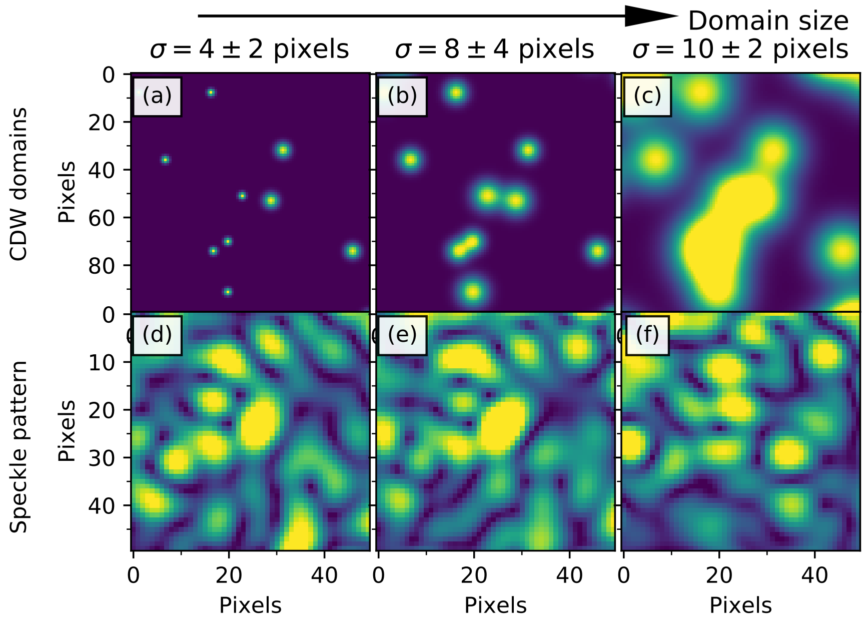

Supplementary Figure 6. Simulation of speckle pattern behavior, based on the uniform expansion of domains about the same locations. (a)-(c) Real space domain configurations. A zoomed area of 100×100 pixels is shown. The average domain size, σ, is doubled from (a) to (b), and (b) to (c). In (c), domains start to merge into each other. (d)-(f) Speckle patterns for the images in the first row obtained from the Fourier transform squared. A zoomed area of 100×100 pixels located 175 pixels away from the peak-center is shown.

Supplementary Figures

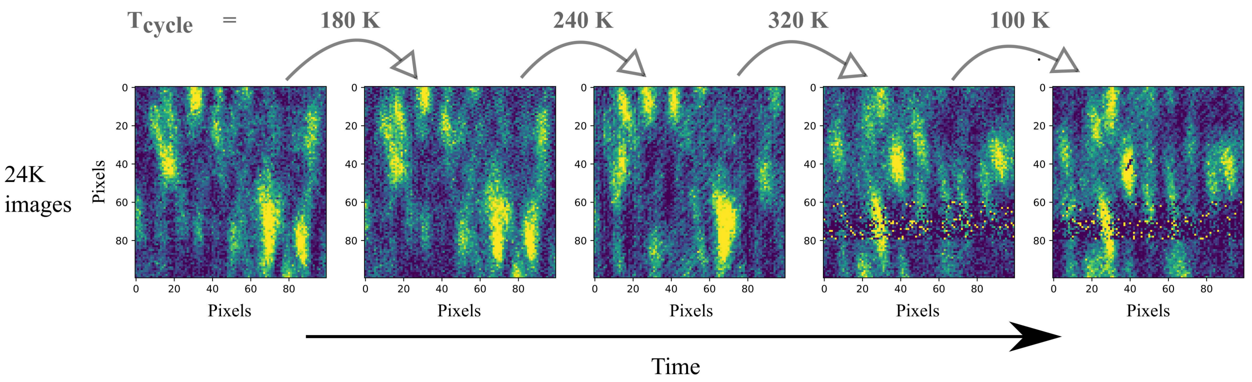

Supplementary Figure 7. Temperature hysteresis of speckle texture in a larger field of view than that presented in Fig. 2(b). CDW speckle images taken at 24 K. The grey arrows indicate the temperature cycling between images. T_{cycle} indicates the highest temperature that sample was brought to during a temperature cycle. Each image presents a 100×100 detector pixel area.

Supplementary References

[1] Michael S Pierce, Rob G Moore, Larry B Sorensen, Stephen D Kevan, Olav Hellwig, Eric E Fullerton, and Jeffrey B Kortright, "Quasistatic X-Ray Speckle Metrology of Microscopic Magnetic Return-Point Memory," Phys. Rev. Lett. **90**, 175502–175504 (2003).

[2] K. Chesnel, J. Nelson, B. Wilcken, and S. D. Kevan, "Mapping spatial and field dependence of magnetic domain memory by soft X-ray speckle metrology," Journal of Synchrotron Radiation **19**, 293–306 (2012).

[3] Karine Chesnel, Alex Safsten, Matthew Rytting, and Eric E Fullerton, "Shaping nanoscale magnetic domain memory in exchange-coupled ferromagnets by field cooling," Nature Communications **7**, 11648 (2016).

[4] X. M. Chen, V. Thampy, C. Mazzoli, A. M. Barbour, H. Miao, G. D. Gu, Y. Cao, J. M. Tranquada, M. P. M. Dean, and S. B. Wilkins, "Remarkable stability of charge density wave order in $La_{1.875}Ba_{0.125}CuO_4$," Phys. Rev. Lett. **117**, 167001 (2016).

[5] V Thampy, XM Chen, Y Cao, C Mazzoli, AM Barbour, W Hu, H Miao, G Fabbris, RD Zhong, GD Gu, *et al.*, "Static charge-density-wave order in the superconducting state of $La_{2-x}Ba_xCuO_4$," Phys. Rev. B **95**, 241111 (2017).

[6] Yimei Zhu, A. R. Moodenbaugh, Z. X. Cai, J. Tafto, M. Suenaga, and D. O. Welch, "Tetragonal-orthorhombic structural modulation at low temperature in $La_{2-x}Ba_xCuO_4$," Phys. Rev. Lett. **73**, 3026–3029 (1994).

[7] Y Horibe, Y Inoue, and Y Koyama, "Direct observation of dynamic local structure in $La_{2-x}Sr_xCuO_4$ around $x = 0.12$," Physical Review B **61**, 11922 (2000).
